# Comparison of NIV-NAVA and NCPAP in facilitating extubation for very preterm infants

**DOI:** 10.1186/s12887-019-1683-4

**Published:** 2019-08-28

**Authors:** Byoung Kook Lee, Seung Han Shin, Young Hwa Jung, Ee-Kyung Kim, Han-Suk Kim

**Affiliations:** 1 Department of Pediatrics, Yonsei University Wonju College of Medicine, Wonju, South Korea; 2 Department of Pediatrics, Seoul National University College of Medicine, Seoul, South Korea; 3 Department of Pediatrics, Seoul National University Children’s Hospital, 101 Daehak-ro, Jongno-gu, Seoul, 110-769 South Korea; 4 Department of Pediatrics, Seoul National University Bundang Hospital, Seongnam, South Korea

**Keywords:** Airway extubation, Continuous positive airway pressure, Neurally adjusted ventilator assist, Noninvasive ventilation, Ventilator weaning

## Abstract

**Background:**

Various types of noninvasive respiratory modalities that lead to successful extubation in preterm infants have been explored. We aimed to compare noninvasive neurally adjusted ventilatory assist (NIV-NAVA) and nasal continuous positive airway pressure (NCPAP) for the postextubation stabilization of preterm infants.

**Methods:**

This retrospective study was divided into two distinct periods, between July 2012 and June 2013 and between July 2013 and June 2014, because NIV-NAVA was applied beginning in July 2013. Preterm infants of less than 30 weeks GA who had been intubated with mechanical ventilation for longer than 24 h and were weaned to NCPAP or NIV-NAVA after extubation were enrolled. Ventilatory variables and extubation failure were compared after weaning to NCPAP or NIV-NAVA. Extubation failure was defined when infants were reintubated within 72 h of extubation.

**Results:**

There were 14 infants who were weaned to NCPAP during Period I, and 2 infants and 16 infants were weaned to NCPAP and NIV-NAVA, respectively, during Period II. At the time of extubation, there were no differences in the respiratory severity score (NIV-NAVA 1.65 vs. NCPAP 1.95), oxygen saturation index (1.70 vs. 2.09) and steroid use before extubation. Several ventilation parameters at extubation, such as the mean airway pressure, positive end-expiratory pressure, peak inspiratory pressure, and FiO_2_, were similar between the two groups. SpO_2_ and pCO_2_ preceding extubation were comparable. Extubation failure within 72 h after extubation was observed in 6.3% of the NIV-NAVA group and 37.5% of the NCPAP group (*P* = 0.041).

**Conclusions:**

The data in the present showed promising implications for using NIV-NAVA over NCPAP to facilitate extubation.

## Background

Invasive mechanical ventilation (MV) is frequently required in preterm infants after birth to maintain adequate alveolar ventilation and effective gas exchange. However, tracheal intubation and MV in preterm neonates can induce ventilator-induced lung injury (VILI) and airway inflammation [1, 2]. Prolonged MV in preterm infants also increases the risk of ventilator-associated pneumonia, increasing the length of hospital stays, mortality, and neurologic impairment [3]. Therefore, noninvasive respiratory modalities have been used in preterm infants to facilitate the transition to spontaneous breathing following extubation [4–7].

Nasal continuous positive airway pressure (NCPAP) maintains functional residual capacity while improving lung compliance and oxygenation. NCPAP has been widely used in the neonatal intensive care unit (NICU) and has proven to be effective in preventing failure of extubation in preterm infants [8]. However, studies have reported that extubation failure rates ranged from 25 to 35% among preterm infants who were given NCPAP after extubation [9, 10]. Nasal intermittent positive pressure ventilation (NIPPV) augments NCPAP by superimposing ventilator inflation on NCPAP [11]. Although synchronized (SNIPPV) or nonsynchronized techniques can be used to supplement the infants’ own breathing efforts, it is likely that more effective support can be achieved with SNIPPV [12, 13]. To date, pneumatic capsules or flow sensors have been used to detect inspiration for synchronization, but some limitations in clinical practice have been reported [14–16].

Neurally adjusted ventilatory assist (NAVA) improves synchrony in patients with respiratory support by detecting the electrical activity of the diaphragm and may offer potential benefits in neonatal ventilation [17–20]. Noninvasive ventilation using NAVA as a triggering modality (NIV-NAVA) could be effective, as demonstrated in adult populations [21, 22]. To date, few studies of NIV-NAVA in preterm infants have been conducted. Patient-ventilator synchrony and effective diaphragmatic unloading were reported in preterm infants during NAVA-derived noninvasive nasal ventilation [23]. Herein, we aimed to compare NIV-NAVA and NCPAP for the postextubation stabilization of very low birth weight infants.

## Methods

This study used a retrospective approach and was approved by the Institutional Review Board of Seoul National University Hospital. The study included preterm infants of less than 30 weeks gestational age (GA) who were admitted to the NICU of the Seoul National University Children’s Hospital (SNUCH) between July 2012 and June 2014 and survived more than 72 h. Infants who were on MV for longer than 24 h and were weaned to NCPAP (Infant Flow system, Viasys, Healthcare, Pennsylvania, United States) or NIV-NAVA (SERVO-I, Maquet Critical Care AB, Solna, Sweden) after extubation were eligible for the study. The size of the Edi catheter used during the study period was 6 Fr/49 cm, which could be used for extremely preterm infants [20]. There were no postmenstrual age (PMA) criteria for the use of NIV-NAVA during the study period if self-respiration was well established in the baby. Infants who had major congenital anomalies or who were intubated for longer than 6 weeks were excluded from the study. The study period was divided into two distinct periods, namely between July 2012 and June 2013 (Period I) and between July 2013 and June 2014 (Period II), because NIV-NAVA was applied at SNUCH beginning in July 2013.

The respiratory severity score (RSS = mean airway pressure (cmH_2_O) x FiO_2_) and oxygen saturation index (OSI = MAP x FiO_2_ × 100 ÷ SpO_2_) were used to compare the pre-extubation respiratory conditions between the two groups [24, 25]. The RSS has been used to predict extubation readiness or the length of mechanical ventilation in preterm infants, and the OSI has been suggested to be a useful measurement to reliably assess the severity of respiratory conditions in preterm infants when the oxygen index is not available [26, 27]. During the study period, extubation was performed if the patient remained stable with a SpO_2_ > 90% for at least 6 h while on the following settings: mean airway pressure (MAP) ≤ 9 cmH_2_O, positive end expiratory pressure (PEEP) ≤ 7 cmH_2_O and fraction of inspired oxygen (FiO_2_) ≤ 40%. In infants who were mechanically ventilated for longer than 15 days, dexamethasone was administered to reduce airway edema. All infants included in the study population were treated with caffeine. A capillary blood gas analysis was performed within 1 h after extubation. Postextubation PEEP was initially set to 5~6 cmH_2_O both in the NCPAP and NIV-NAVA groups, and was then adjusted within a range of 4~8 cmH_2_O according to the clinician’s discrimination. The NAVA level was initially set to 1.0~1.5 cmH_2_O/μV and adjusted to obtain pCO_2_ < 70 mmHg. In both ventilation strategies, binasal prongs and masks were used alternatively every 24 h to minimize nasal injury.

The primary outcome of the study was extubation failure within 72 h after extubation, which was defined according to a set of conditions for reintubation and the reapplication of MV [28]. Infants with severe apnea requiring positive pressure ventilation (PPV), ≥ 4 apneic episodes per hour needing moderate stimulation, FiO_2_ > 60%, or uncompensated respiratory acidosis (pH < 7.25) were reintubated during the study period. Backup ventilation at a rate of 30/min and pressure of 10–15 cmH_2_O above PEEP was applied if Edi was absent or apnea occurred for more than 5–10 s and the upper pressure limit was set to 20–25 cmH_2_O [23].

All statistical analyses were performed with STATA 11.0 (Stata Corp, College Station, TX, USA) using the independent t-test for continuous variables and the χ^2^-test and Fisher’s exact test for categorical variables. For all statistical analyses, *P* < 0.05 was considered statistically significant.

## Results

**Fig. 1:**
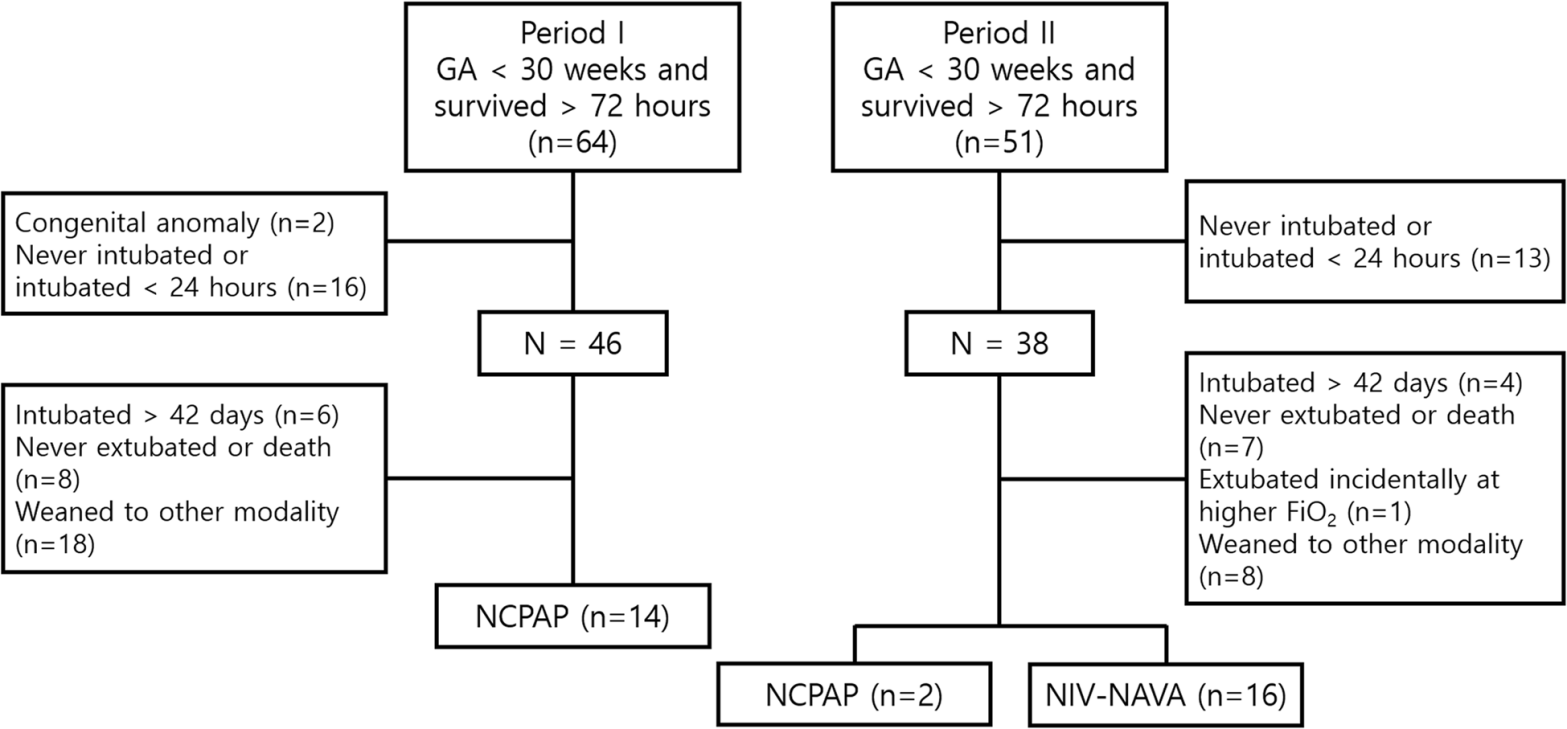
Selection of the study population during the study period

A total of 64 infants in Period I and 51 infants in Period II who were born at less than 30 weeks of gestation and survived greater than 72 h were admitted (Fig. 1). Two infants from Period I were excluded: one infant had Beckwith-Wiedemann syndrome, and the other infant had Galen malformation of the brain. Sixteen infants in Period I and 13 infants in Period II who were never intubated or intubated less than 24 h were also excluded. After excluding infants who had been intubated for greater than 6 weeks, those who were never extubated or died before discharge, and those who were weaned to other modalities, such as heated and humidified high flow nasal cannula (HHHFNC), there were 14 infants who were weaned to NCPAP during Period I and 16 infants who were weaned to NIV-NAVA during Period II. The 2 infants who were weaned to NCPAP during Period II were categorized as the NCPAP group with the infants from Period I.

The GA and birth weight of the NIV-NAVA group and NCPAP group were not significantly different (27^+ 1^ vs. 26^+ 5^ weeks and 875 vs. 845 g, respectively) (Table 1). The incidence of RDS, maternal histologic chorioamnionitis and antenatal steroid use were also not significantly different between the two groups. At the time of extubation, PMA and weight exhibited no significant differences between the NIV-NAVA group and NCPAP group (30 vs. 29^+ 4^ weeks and 1045 vs. 1205 g, respectively) (Table 2). No differences in RSS (NIV-NAVA 1.65 vs. NCPAP 1.95), OSI (1.70 vs. 2.09) or steroid use were noted before extubation. Several ventilation parameters at extubation, such as MAP, PEEP, PIP peak inspiratory pressure (PIP), and FiO_2_, were similar between the two groups. SpO_2_ and pCO_2_ preceding extubation were also comparable.

**Table 1:** Demographics of the study population

	NIV-NAVA (*n* = 16)	NCPAP (*n* = 16)	*P* value
GA (weeks)	27^+ 1^ (26^+ 5^, 27^+ 6^)	26^+ 5^ (25^+ 4^, 27^+ 6^)	0.317
Birth weight (grams)	875 (677.5, 1145)	845 (700, 1030)	0.777
Male	11 (68.8)	7 (43.8)	0.143
C/S	8 (50.0)	7 (43.8)	0.500
Multiple births	12 (75.0)	10 (62.5)	0.352
PIH	4 (25.0)	1 (6.25)	0.166
hCAM	5 (31.3)	10 (62.5)	0.078
PPROM	7 (43.8)	6 (37.5)	0.500
Antenatal steroid	7 (43.8)	12 (75.0)	0.074
1-min AS	3 (2, 5)	3.5 (2, 4.5)	0.802
5-min AS	5.5 (4, 7)	7 (6, 7)	0.122
RDS	14 (87.5)	16 (100)	0.242
PDA	12 (75.0)	7 (73.3)	0.618

Values are presented as the median (interquartile range) or n (%)

*NIV-NAVA* Noninvasive neurally adjusted ventilatory assist, *NCPAP* Nasal continuous positive airway pressure, *GA* Gestational age, *C/S* Cesarean section, *PIH* Pregnancy induced hypertension, *hCAM* Histologic chorioamnionitis, *PPROM* Preterm premature rupture of membrane, *AS* Apgar score, *RDS* Respiratory distress syndrome, *PDA* Patent ductus arteriosus

**Table 2:** Clinical characteristics at the time of extubation

	NIV-NAVA (*n* = 16)	NCPAP (*n* = 16)	*P* value
PMA at extubation (weeks)	30 (28^+ 6^, 31^+ 4^)	29^+ 4^ (27^+ 3^, 30^+ 4^)	0.282
Weight at extubation (grams)	1045 (800, 1325)	1025 (905, 1190)	0.651
Pre-extubation			
Ventilator duration (days)	21.5 (11.5, 27)	9.5 (4.5, 34.5)	0.365
Systemic steroid use	7 (43.8)	5 (31.3)	0.358
RSS	1.65 (1.49, 2.28)	1.95 (1.68, 2.32)	0.317
OSI	1.70 (1.53, 2.39)	2.09 (1.76, 2.51)	0.274
MAP (cmH_2_O)	7 (7, 7.5)	8 (7, 8)	0.212
PEEP (cmH_2_O)	5 (5, 5)	5 (5, 6)	0.531
PIP (cmH_2_O)	13 (12, 14)	15 (12, 16)	0.180
FiO_2_ (%)	0.24 (0.21, 0.31)	0.25 (0.21, 0.30)	0.700
pCO_2_ (mmHg)	53.2 (45.0, 58.4)	49.1 (43.7, 65.3)	0.970
SpO_2_ (mmHg)	95.5 (94, 98.5)	96 (93.5, 97)	0.760

Values are presented as the median (interquartile range) or n (%)

*NIV-NAVA* Noninvasive neurally adjusted ventilatory assist, *NCPAP* Nasal continuous positive airway pressure, *PMA* Postmenstrual age, *RSS* Respiratory severity score, *OSI* Oxygen saturation index, *MAP* Mean airway pressure, *PEEP* Positive end-expiratory pressure, *PIP* Peak inspiratory pressure

**Fig. 2:**
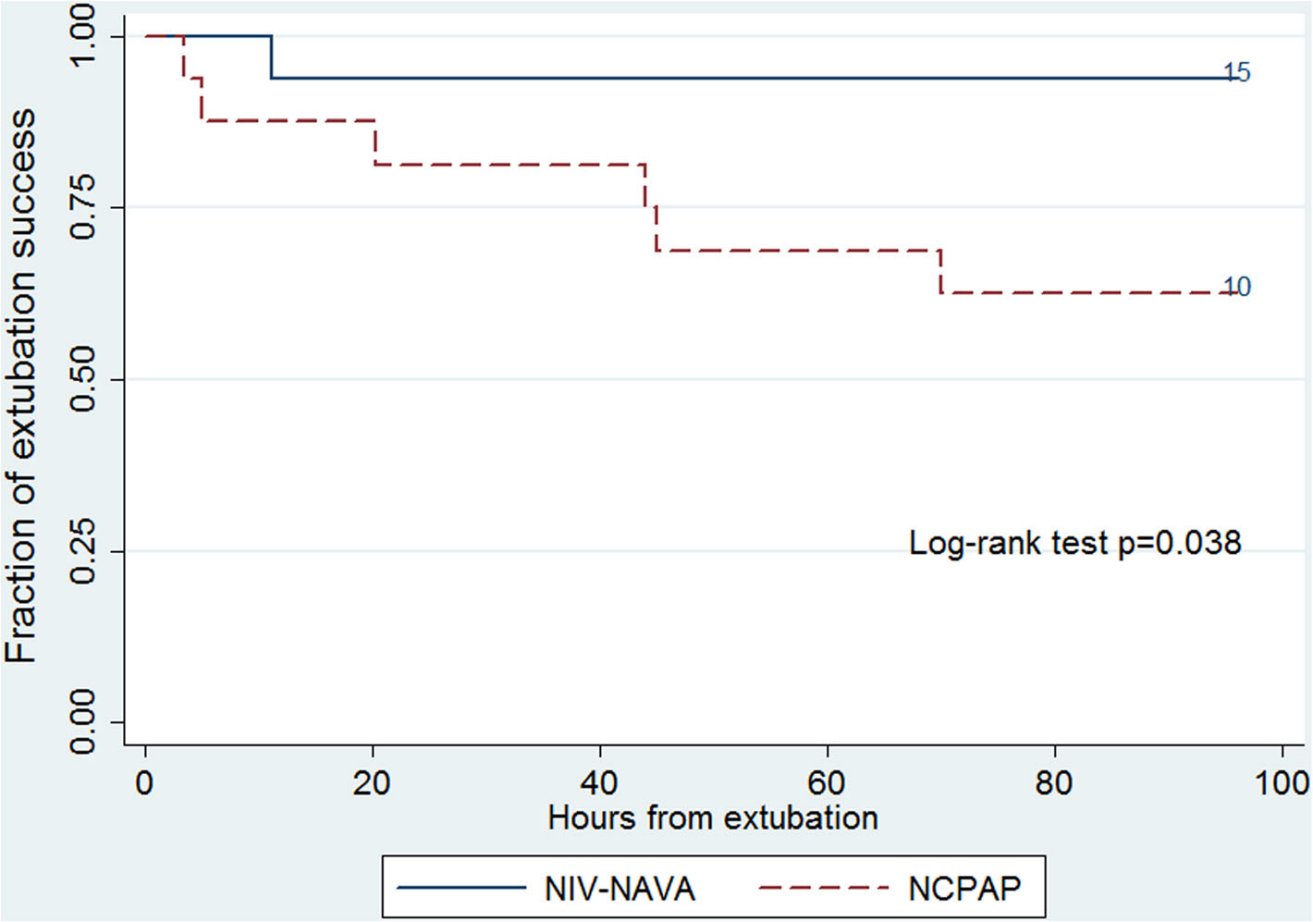
Kaplan-Meier estimates for extubation success by post-extubation modality

Extubation failure within 72 h after extubation was ascertained in 1 (6.3%) infant in the NIV-NAVA group and 6 (37.5%) infants in the NCPAP group (*P* = 0.041) (Table 3). One infant in the NIV-NAVA group was reintubated 11 h after extubation because of severe apnea requiring PPV. In the NCPAP group, 3 infants were reintubated before 24 h after extubation, 2 infants were reintubated 24–48 h after extubation and one infant was reintubated 70 h after extubation (Fig. 2). Three infants were reintubated because of severe apnea requiring PPV, two infants due to uncompensated respiratory acidosis (pH < 7.25) with pCO_2_ > 70 mmHg and one infant due to ≥4 apneic episodes per hour needing moderate stimulation. The use of other respiratory support parameters after extubation, such as PEEP and FiO_2_, were comparable between the NCPAP and NIV-NAVA groups with similar pCO_2_ and SpO_2_. Among those who were reintubated in the study, GA at birth was 26.4 weeks in the NIV-NAVA group and 25.9 (25.3–28.1) weeks in the NCPAP group. In the univariate logistic regression analysis, GA at extubation and the duration of invasive ventilation before extubation were not associated with reintubation (data not shown).

**Table 3:** Post-extubation status of the study population

	NIV-NAVA (*n* = 16)	NCPAP (*n* = 16)	*P* value
PEEP (cmH_2_O)	6 (5.5, 6)	6 (5, 7)	1.000
FiO_2_ (%)	0.30 (0.27, 0.35)	0.25 (0.21, 0.33)	0.109
pCO_2_ (mmHg)	48.5 (44.3, 53.6)	49.7 (40.7, 62.1)	0.695
SpO_2_ (mmHg)	96 (93, 97)	96.5 (94, 98)	0.597
Extubation failure ≤72 h	1 (6.3)	6 (37.5)	0.041

Values are presented as the median (interquartile range) or n (%). Post-extubation status was checked 1 h after extubation

*NIV-NAVA* Noninvasive neurally adjusted ventilatory assist, *NCPAP* Nasal continuous positive airway pressure, *PMA* Postmenstrual age, *RSS* Respiratory severity score, *OSI* Oxygen saturation index, *MAP* Mean airway pressure, *PEEP* Positive end-expiratory pressure, *PIP* Peak inspiratory pressure

No differences were noted between the two groups regarding the other clinical outcomes, including the development of moderate to severe bronchopulmonary dysplasia (BPD) (Table 4).

**Table 4:** Clinical outcomes of the study population

	NIV-NAVA (*n*=16)	NCPAP (*n* = 16)	*P* value
Moderate to severe BPD	10 (62.5)	9 (60.0)	0.589
NEC ≥ stage 2	2 (12.5)	5 (33.3)	0.170
Retinopathy of prematurity	4 (25.0)	6 (40.0)	0.306
IVH ≥ grade 2	2 (12.5)	1 (6.7)	0.525
Periventricular leukomalacia	1 (6.3)	0 (0)	0.516

Values are presented as the median (interquartile range) or n (%)

*NIV-NAVA* Noninvasive neurally adjusted ventilatory assist, *NCPAP* Nasal continuous positive airway pressure, *BPD* Bronchopulmonary dysplasia, *NEC* Necrotizing enterocolitis, *ROP* Retinopathy of prematurity, *IVH* Intraventricular hemorrhage

## Discussion

Extubation failure is often observed in preterm infants because the chest wall and upper airway collapses easily and diaphragmatic strength is poor [29, 30]. The present study revealed that NIV-NAVA facilitated extubation better than NCPAP. Following a period of endotracheal intubation and IPPV, NCPAP is effective for preventing extubation failure in preterm infants [8]. This technique appears to improve lung function and reduce apnea and may therefore play a role in facilitating extubation in this population. However, certain populations among preterm infants who were subject to NCPAP experienced extubation failure [6, 31–33].

NIPPV augments NCPAP by delivering ventilator breaths via nasal prongs or a mask. Although it did not improve ventilation in infants who were able to maintain their own ventilation on NCPAP, in infants with a higher baseline PaCO_2_, ventilation was more effectively increased by NIPPV than NCPAP [34]. Severe apnea and increased PaCO_2_ were the most common causes of failure in infants receiving NCPAP, and NIPPV achieved a comparative reduction in extubation failure in preterm infants. A recent meta-analysis demonstrated that the incidence of extubation failure and the need for reintubation within 48 h to 1 week was reduced by NIPPV in preterm infants [12]. However, synchronization and the device used to deliver PPV may be important parameters in NIPPV [13].

NAVA has been applied in clinical practice during the last decade, but studies have rarely involved neonates, especially the preterm infant population. However, a recent study demonstrated the effectiveness and feasibility of NAVA in this population [19]. Noninvasive support via NAVA improved patient-ventilator synchrony by reducing trigger delay and the number of asynchrony events [35]. Previously, we reported that NAVA improved patient-ventilator synchrony and diaphragmatic unloading in preterm infants during noninvasive nasal ventilation compared with pressure support mode [23]. A recent physiologic study performed by Gibu et al. compared NIV-NAVA and NIPPV and demonstrated that peak inspiratory pressure and FiO_2_ were lowered in NIV-NAVA than in NIPPV [36]. Furthermore, both infant movement and caretaker’s work were lowered in NIV-NAVA, suggesting that NIV-NAVA was more effective than NIPPV at increasing infant comfort. Because it has excellent synchronization, NIN-NAVA could serve as a substitute for NCPAP to facilitate extubation in preterm infants. Most cases of reintubation in this study were the result of severe apnea or uncompensated hypercapnia. When compared to NCPAP, apnea and hypercapnia were more preventable in NIPPV by generating higher airway pressure to prevent obstructive apnea and triggering sigh in preterm infants [37, 38]. Although NIV-NAVA seemed to improve ventilator synchrony and diaphragmatic unloading during noninvasive ventilation compared to other NIPPV, there was no evidence that NIV-NAVA is superior to other NIPPV modalities after extubation [23, 39].

Even though there could be concerns regarding the size of the baby when using NIV-NAVA, many studies showed NIV-NAVA was feasible in extremely preterm infants [23, 39]. In the present study, NIV-NAVA was also found to be feasible in babies as small as 660 g at extubation or 700 g at birth who were successfully weaned to NIV-NAVA at PMA 28 weeks. A baby who was 500 g at birth was also successfully weaned to NIV-NAVA at 770 g. Moreover, Edi catheters can efficiently serve as a feeding tube in these babies and thus an additional feeding tube did not need to be inserted for enteral feeding. NEC was comparable in both groups and there were no intestinal perforations or air leaks after the infants were weaned to NIV-NAVA or NCPAP. Although the rates of neonatal complications are lower in noninvasive versus invasive MV, safety must be considered. Previously, it was suggested that neonates who were mechanically ventilated with either a face mask or nasal prongs had an increased risk of gastrointestinal perforations. However, recent data has shown that NIPPV does not appear to be associated with increased gastrointestinal side effects, and the risk of air leaks was lower in NIPPV than in NCPAP [40]. No differences in the development of air leaks and NEC were observed between the two groups in the present study.

There are some limitations to the present study. This study was a retrospective study with a small sample size, thus making it difficult to draw robust conclusions. There also was a period of overlap when both NIV-NAVA and NCPAP were used as weaning modalities. The study population was highly selected because we analyzed only 50% of the preterm infants born at < 30 weeks of gestation who were intubated for more than 24 h and were extubated thereafter during the study period. Furthermore, the duration of ventilation seemed to be shorter in the NCPAP group, although this result was not statistically significant. While the sample size may have been too small to fully elucidate this difference, a logistic regression analysis for reintubation was performed ad hoc and showed that the duration of ventilation before extubation was not associated with reintubation (data not shown). The criteria for extubation were well-defined in our unit, and the pre-extubation conditions in both groups including the PMA at extubation, RSS, OSI and the ventilation settings were comparable in the present study. Despite these limitations, this is the first study to compare the clinical responses between NIV-NAVA and NCPAP when used to facilitate extubation in preterm infants.

## Conclusions

The data in the present study were not robust enough to be conclusive due to small sample size, but showed promising implications for using NIV-NAVA over NCPAP to facilitate extubation. NIV-NAVA could be an effective modality for synchronized noninvasive ventilation following successful extubation from MV in preterm infants.

## Data Availability

The dataset generated or analyzed during this study can be made available to interested researchers by the authors of this article upon reasonable request.

## Abbreviations

HHHFNC: Humidified high flow nasal cannula
MAP: Mean airway pressure
MV: Mechanical ventilation
NAVA: Neurally adjusted ventilatory assist
NCPAP: Nasal continuous positive airway pressure
NICU: Neonatal intensive care unit
NIPPV: Nasal intermittent positive pressure ventilation
NIV-NAVA: Non-invasive ventilation using NAVA
OSI: Oxygen saturation index
PEEP: Positive end expiratory pressure
PPV: Positive pressure ventilation
RSS: Respiratory severity score
SNIPPV: Synchronized Nasal intermittent positive pressure ventilation
VILI: Ventilator-induced lung injury
